# Correction: Rydén et al. Physiological and Clinical Responses in Pigs in Relation to Plasma Concentrations during Anesthesia with Dexmedetomidine, Tiletamine, Zolazepam, and Butorphanol. *Animals* 2021, *11*, 1482

**DOI:** 10.3390/ani11123474

**Published:** 2021-12-06

**Authors:** Anneli Rydén, Marianne Jensen-Waern, Görel Nyman, Lena Olsén

**Affiliations:** Department of Clinical Sciences, Swedish University of Agricultural Sciences, SE-750 07 Uppsala, Sweden; marianne.jensen-waern@slu.se (M.J.-W.); gorel.nyman@slu.se (G.N.); lena.olsen@slu.se (L.O.)

**Text Correction**

There were several errors in the original article [1]:

1. In the sixth line of the abstract, instead of “The anesthesia was induced by an intramuscular injection of dexmedetomidine, tiletamine zolazepam, and butorphanol in 12 pigs”. It should be “The anesthesia was induced by an intramuscular injection of dexmedetomidine, tiletamine-zolazepam, and butorphanol in 12 pigs”.
2. In Section 3.3, on page 6, instead of “In all pigs, the average Cmax for all drugs was 12 (range 10–13) after administration of the IM induction drug combination”, it should be “In all pigs, the average Cmax for all drugs was reached 12 (range 10–13) min after administration of the IM induction drug combination”.
3. In Section 3.3, on the bottom line of page 6, instead of “IV (Table 2)”, it should be “IV (Figure 2)”.

**Figure/Table Legend**

In the original article, there were mistakes in the legend for Table 3 and Figure 2. The correct legends appears below. 

**Table 3.** Drug analysis data.

**Figure 2.** Profiles of log plasma concentrations for each drug over time for short-term anesthesia induced by intramuscular administration of dexmedetomidine 0.025 mg/kg, tiletamine 2.5 mg/kg, zolazepam 2.5 mg/kg, and butorphanol 0.1 mg/kg in pigs (*n* = 7). Group S (dotted lines *n* = 3) received a single dose at time zero. Group R (solid lines *n* = 4) also received one-third of the initial dose intravenously at 60 min. Time to first spontaneous movement is indicated with a dotted arrow for Group S and a solid arrow for Group R.

**Error in Table**

In the original article, there was a mistake in Tables 2 and 4 as published.

1. On page 8, in the third line of the footer of Table 4, instead of “and group Ralso”, it should be “and group R also”.
2. The footnote under Table 4 should be placed under Table 2.

The corrected Table 2 and Table 4 appear below. The authors apologize for any inconvenience caused and state that the scientific conclusions are unaffected. The original article has been updated.

We apologize for any inconvenience caused to the readers by this change. The change does not affect the scientific results. The original article has been updated.

## Figures and Tables

**Table 2:** Observed clinical responses in relation to plasma concentrations.

			Concentration Range (ng/mL) Group S and R			
Noted Observations	Time (min) from Injection (IM)		Dexmedetomidine	Tiletamine	Zolazepam	Butorphanol
	S	R				
Lateral recumbency	2–4		<1.59–3.56	<126–268	<106–789	<5.22–15.60
Unconsciousness	5–15		1.99–6.00	169–337	737–1330	8.86–19.20
Last sample before response to noxious stimulus	60–120	120	2.01–3.48	115–158	520–1180	6.74–11.60
Palpebral reflexes	60–70	126–180	1.69–2.38	101–129	525–782	6.80–10.50
Response to noxious stimulus	70–140	126–180	1.69–2.38	101–129	525–782	6.80–10.50
First movement	70–140	126–180	1.69–1.97	106–123	444–713	6.47–9.42
Standing	120–180	175–240	1.14–2.01	73.7–109	250–678	4.74–8.70

Time range in min and the corresponding plasma concentration range for each drug and noted observation during the short-term anesthesia induced by dexmedetomidine 0.025 mg/kg, tiletamine 2.5 mg/kg, zolazepam 2.5 mg/kg, and butorphanol 0.1 mg/kg after intramuscular (IM) administration in growing pigs single (S) (*n* = 6) and repeated (R) (*n* = 6). All pigs received a single dose at time zero, and group R also received one-third of the initial dose intravenously at 60 min. All blood samples were taken in relation to the noted observation except for lateral recumbency, which was taken at 5 min after induction. Plasma concentrations were available for seven pigs.

**Table 4:** Pharmacokinetic parameters.

	IM Group S (*n* = 3)			IV Group R (*n* = 4)		
	t_½_ (min)	t_max_ (min)	C_max_ (ng/L)	t_½_ (min)	cI (L/kg/min)	Vd (L/kg)
Dexmedetomidine	125 ± 37	13 ± 3	5.6 ± 3.2	117 ± 24	0.012 ± 0.004	1.7 ± 0.5
Tiletamine	90 ± 12	10 ± 5	342 ± 152	80 ± 13	0.050 ± 0.010	5.8 ± 0.9
Zolazepam	72 ± 6	12 ± 3	1372 ± 438	76 ± 13	0.009 ± 0.002	1.0 ± 0.2
Butorphanol	97 ± 11	13 ± 3	21 ± 9	101 ± 16	0.012 ± 0.002	1.6 ± 0.4

## References

[1] Rydén A., Jensen-Waern M., Nyman G., Olsén L. (2021). Physiological and Clinical Responses in Pigs in Relation to Plasma Concentrations during Anesthesia with Dexmedetomidine, Tiletamine, Zolazepam, and Butorphanol. Animals.

